# Health system learning with Indigenous communities: a study protocol for a two-eyed seeing review and multiple case study

**DOI:** 10.1186/s12961-022-00873-8

**Published:** 2022-06-16

**Authors:** Crystal Milligan, Rosa Mantla, Grace Blake, John B. Zoe, Tyanna Steinwand, Sharla Greenland, Susan Keats, Sara Nash, Kyla Kakfwi-Scott, Georgina Veldhorst, Angela Mashford-Pringle, Suzanne Stewart, Susan Chatwood, Whitney Berta, Mark J. Dobrow

**Affiliations:** 1 Institute of Health Policy, Management and Evaluation, Dalla Lana School of Public Health, University of Toronto, 155 College Street, Toronto, ON Canada; 2 Elder, Behchokǫ̀, NT Canada; 3 Elder, Tsiigehtchic, NT Canada; 4 Tłı̨chǫ Government, Behchokǫ̀, NT Canada; 5 School of Public Health, University of Alberta, Edmonton, AB Canada; 6 Gwich’in Tribal Council, Inuvik, NT Canada; 7 Tłı̨chǫ Community Services Agency, Behchokǫ̀, NT Canada; 8 Department of Health and Social Services, Government of Northwest Territories, Yellowknife, NT Canada; 9 Northwest Territories Health and Social Services Authority, Government of Northwest Territories, Yellowknife, NT Canada; 10 Waakebiness-Bryce Institute for Indigenous Health, Dalla Lana School of Public Health, University of Toronto, Toronto, ON Canada

**Keywords:** Organizational learning, Learning health systems, Indigenous health, Knowledge, Evidence, Two-eyed seeing, Canada

## Abstract

**Background:**

It is well documented that Canadian healthcare does not fully meet the health needs of First Nations, Inuit or Métis peoples. In 1996, the Royal Commission on Aboriginal Peoples concluded that Indigenous peoples’ healthcare needs had to be met by strategies and systems that emerged from Indigenous worldviews and cultures. In 2015, the Truth and Reconciliation Commission also called on health organizations to learn from Indigenous knowledges and integrate Indigenous worldviews alongside biomedicine and other western ways of knowing. These calls have not yet been met. Meanwhile, the dynamic of organizational learning from knowledges and evidence within communities is poorly understood—particularly when learning is from communities whose ways of knowing differ from those of the organization. Through an exploration of organizational and health system learning, this study will explore how organizations learn from the Indigenous communities they serve and contribute to (re-)conceptualizing the learning organization and learning health system in a way that privileges Indigenous knowledges and ways of knowing.

**Methods:**

This study will employ a two-eyed seeing literature review and embedded multiple case study. The review, based on Indigenous and western approaches to reviewing and synthesizing knowledges, will inform understanding of health system learning from different ways of knowing. The multiple case study will examine learning by three distinct government organizations in Northwest Territories, a jurisdiction in northern Canada, that have roles to support community health and wellness: Tłı̨chǫ Government, Gwich’in Tribal Council, and Government of Northwest Territories. Case study data will be collected via interviews, talking circles, and document analysis. A steering group, comprising Tłı̨chǫ and Gwich’in Elders and representatives from each of the three partner organizations, will guide all aspects of the project.

**Discussion:**

Examining systems that create health disparities is an imperative for Canadian healthcare. In response, this study will help to identify and understand ways for organizations to learn from and respectfully apply knowledges and evidence held within Indigenous communities so that their health and wellness are supported. In this way, this study will help to guide health organizations in the listening and learning that is required to contribute to reconciliation in healthcare.

## Background

The overarching aim of this study is to improve understanding of how health systems learn from the knowledges and evidence held within Indigenous communities.

It is well documented that Canadian healthcare does not meet the health needs of First Nations, Inuit or Métis peoples, hereafter collectively referred to as Indigenous peoples [1–4]. In fact, health organizations perpetuate the preservation of colonial structures [1, 4, 5], culturally incompetent services [3], and situations where some Indigenous people wait until advanced stages of disease before seeking care, or prefer not to seek care at all [2]. A cultural construct, mainstream healthcare reflects western, individualistic, biomedical values and tends not to support traditional Indigenous health beliefs and ways of knowing that take a more wholistic view of health as harmony between individuals and their family, community, environment and spirit [1, 3, 4]. The Royal Commission on Aboriginal Peoples concluded in 1996 that Indigenous peoples’ healthcare needs had to be met by strategies and systems that emerged from Indigenous worldviews and cultures [1]. In 2015, the Truth and Reconciliation Commission also called on organizations to learn from Indigenous knowledges and integrate Indigenous worldviews alongside biomedicine and other western ways of knowing [4]. These calls have not yet been met.

Contemporaneously with this push to improve how health systems learn from Indigenous knowledges, the concept of a *learning health system* has been enthusiastically promoted in western academic literature since it was first proposed as a concept in 2006 by the National Academy of Medicine (formerly the Institute of Medicine) [6]. Though frequently invoked, the concept is rarely defined in healthcare literature. Even so, there has been more than a decade of progressive interest in advancing our understanding of learning health systems, conceptualizations of which vary considerably. With no consensus on a definition, the term is inconsistently used to refer to initiatives at the micro, meso and macro levels of the health system, running the gamut from sub-organizational tools for learning [7] to organizational [8] or system-wide [9] learning. Subject to definitional clarity, it is questionable whether more than a handful of learning health systems—if any—exist at the meso or macro level [10–12].

While the communities a healthcare organization serves represent a relevant source of knowledges that are essential to improve services, how organizations and systems learn from or with communities is understudied [13]. Instead, the emergent learning health systems literature is dominated by discussions of electronic health records as a primary source of evidence [14]. While such technology offers useful tools for widespread transmission of quantified, explicit knowledge, it fails to capture the tacit understandings of culture, identity and ideology that are so important to improve services. Moreover, this rather narrow orientation toward clinical research and health service delivery neither adequately captures the breadth and depth of relevant theory from other research traditions (e.g. Indigenous research, organizational learning, complexity science) nor promotes a model that prioritizes nonclinical or tacit forms of knowledge.

Despite these concerns, there has been more than a decade of progressive interest in advancing the concept of a learning health system. Health systems around the world are increasingly aiming to create the conditions for learning health systems, though conceptualizations vary considerably and are often detached from organizational learning theory [13]. Thus, there is an important and timely opportunity to unpack diverse understandings of health system learning such that Indigenous knowledges can play a more integral role in theory building and the evolution of health systems. Indeed, recent research explicitly recognizes that Indigenous conceptualizations of the characteristics of learning health systems are needed [9]. Indigenous and western definitions of health are not mutually exclusive, and their integration can broaden our understanding of health and healthcare [15].

As health system learning from knowledges and evidence within Indigenous communities has not been examined before, nor have Indigenous peoples’ perspectives previously been privileged or applied to enhance understanding of learning health systems, some readers may find it helpful to review Table 1, which briefly outlines the definitions or framing of several key terms used in this study protocol.

**Table 1:** Notes on terminology

Indigenous *Indigenous* is a collective name that refers to all the original peoples of a given region in Canada or other countries, unlike groups that have settled, occupied or colonized the region. Indigenous peoples living in Canada comprise three broad groups: First Nations, Inuit and Métis. Where possible, we use the name according to which an individual or group self-identifies. Otherwise, we use the term *Indigenous* to collectively refer to these three groups. This is meant to acknowledge similarities in the colonial experience, not to deny the plurality of rich cultures and histories among them. The term *Aboriginal* is used only in a historical context, or regarding policy or report titles
Western *Western* may be understood here as referring to the values, social norms, customs, political systems and other elements of society that originated in or are otherwise associated with Europe. In the Canadian context, *western* may be used interchangeably with *mainstream* to denote the dominance of Eurocentric, white cultural systems
Indigenous and western ways of knowing Indigenous and western ways of knowing differ in their ways of understanding the world [16–18]. Indigenous ways focus on understanding that is wholistic (where the intentional use of the “w” refers to the whole person). Western ways are inclined toward simplification by reductionism and compartmentalization. However, there is diverse variation within Indigenous and western ways of knowing alike
Knowledge *Knowledge* can comprise any facts, ideas, practice, experience or worldview. Reference to plural knowledges not only distinguishes Indigenous from western knowledge systems, but also respectfully acknowledges the multiplicity of ways of knowing that exist among Indigenous peoples as well as non-Indigenous peoples [19]
Evidence *Evidence* is broadly defined as knowledge in context, including all knowledge acquired through the senses, spirit and relationships [17]
Elder Different communities and cultures have different ways of defining what makes an *Elder*. In general, Indigenous Elders hold deep knowledge in areas such as ceremony, traditional teachings and history. They possess traits such as wisdom and leadership, serve as teachers and stewards of knowledge and are foundational to community well-being [20]. Status as an Elder is determined by the community and is not dependent on age
Community *Community* is broadly defined as a community of “the people” such as a town or First Nations, Inuit or Métis group. The community represents a social, political and knowledge context in which organizations are embedded or with which they interact, including established norms and worldviews
Organization *Organization* refers to organizations with a mandate to support the health and wellness of the communities they serve (such as governments and health service organizations or agencies)
Organizational learning and the learning organization One aim of this research is to contribute to conceptualizations of a learning organization. Notably, we do not define the learning organization as equivalent to organizational learning. In this protocol, we position *organizational learning* as the process and *learning organization* as the product or “doer” of organizational learning
Learning health system One aim of this research is to contribute to conceptualizations of a learning health system. In this protocol, we broadly and provisionally conceptualize the *learning health system* as an arrangement of many interconnected dimensions and actors with shared purpose to support people’s health

## Research questions

Through an exploration of organizational learning that privileges Indigenous knowledges and ways of knowing, this study will contribute to a more robust and sophisticated understanding of learning organizations and learning health systems, including why, how and what they learn from the knowledges and evidence held within Indigenous communities.

This study will be guided by three research questions that relate to organizational learning, the learning organization and the learning health system, respectively.

1. How and under what conditions do organizations learn from the knowledges and evidence within Indigenous communities that they serve?
2. How might the learning organization be (re-)conceptualized to reflect Indigenous knowledges and ways of knowing?
3. How might a learning health system be conceptualized, privileging Indigenous knowledges and ways of knowing?

## Methods

### Setting

This study was borne out of personal relationships in Northwest Territories (NT), a jurisdiction in northern Canada. The lead author is a lifelong settler Canadian (non-Indigenous) resident of NT, born and raised in Sǫ̀mba K'è, also known as Yellowknife, in Chief Drygeese Territory (Treaty 8). The idea for this research took shape through learning and discussion with other NT residents and organizations about what kind of northern-led research would most benefit their health systems and communities.

The study will, therefore, be carried out in NT. In NT, residents experience poorer health than Canadians in all other jurisdictions except Nunavut, and the health of Indigenous residents is consistently worse than among non-Indigenous residents [21]. Of fewer than 45,000 people, 50.2% self-identifies as Indigenous, a group that comprises First Nations, Inuit and Métis [22]. Many Indigenous residents maintain traditional lifestyles with strong connections to the land. Nearly half the territorial population lives in the capital, Yellowknife, where there is the greatest range of health services, including the territorial hospital, the largest healthcare facility in NT albeit with limited specialty care. In Yellowknife, 76% of the population self-identify as non-Indigenous, including a diversity of ethnicities [22]. This contrasts with the remaining 32 communities in the territory, categorized as *small communities* or *regional centres*, where a median of 89% of residents self-identifies as Indigenous [22].

### Research partners

This study is funded by a Canadian Institutes of Health Research grant (FRN 169070). Two Indigenous governments—the Gwich’in Tribal Council (GTC) and Tłı̨chǫ Government (TG)—and three entities within the territorial government—the NT Department of Health and Social Services (DHSS), NT Health and Social Services Authority (NTHSSA) and Tłı̨chǫ Community Services Agency (TCSA)—are partnered with the University of Toronto on this project. A steering group of Indigenous Elders and representatives from each of the partner organizations has been established to guide and collaborate with the university team, which comprises First Nations and settler Canadian scholars. These scholars commit to listening and learning while upholding the values, knowledges and practices of all partners and local communities [23].

### Conceptual framework

A core principle of this study will be commitment to two-eyed seeing. Defined as a co-learning journey that values both Indigenous and western ways of thinking [24, 25], two-eyed seeing allows for reflexive consideration of the merits, limitations and challenges of different knowledge systems [25]. The Tłı̨chǫ people living in present-day NT have a similar principle of being “strong like two people”, or learning to simultaneously navigate Indigenous and non-Indigenous worlds [18]. Though these terms may be used interchangeably throughout this protocol, the Tłı̨chǫ term is preferred when speaking specifically about the NT context.

Indigenous ways of knowing are absent as yet in scholarly discussions of health system learning, but many Indigenous scholars have already made substantial contributions that should be considered as conceptualizations of a learning health system evolve. Notably, Mi’kmaw educator Battiste’s scholarship on learning [26], Opaskwayak Cree scholar Wilson’s discussions of knowledge [17], and the concept of ethical space as developed by Ermine, a member of Sturgeon Lake First Nation [27], have been particularly influential in the preliminary framing of this study. The depth and breadth of the conceptual framework will grow as knowledge holders add their insights over the course of research.

### Timelines and approach

Despite administrative and logistical delays related to the COVID-19 pandemic, it is anticipated that data collection will be initiated in 2022, with data analysis, reporting and dissemination of findings completed by 2025.

We will take a collaborative approach, privileging Gwich’in and Tłı̨chǫ knowledges throughout the research. Collaborative planning with organizational and community partners, including Elders, has been ongoing since 2018, when conversations to understand their needs and interests in the context of this proposed research began. These conversations will be ongoing throughout the project.

Considering public health emergency measures related to the COVID-19 pandemic, we will maintain a flexible methodological stance to data collection. This will also allow the study to adapt as needed to feedback without shifting or negatively impacting the research questions, direction or rigour of research.

The study consists of a three-phase study design, including a two-eyed seeing review, a multiple case study and integration of findings. The first and second phases may run concurrently within the limits of any public health measures that restrict in-person gatherings and engagement as part of the COVID-19 pandemic response.

### Phase 1: Two-eyed seeing review (TESR)

We will develop and conduct a TESR. Consistent with two-eyed seeing and the Tłı̨chǫ principle of “strong like two people”, this review will be based on both Indigenous and western approaches to review and synthesize knowledges. We will draw on our research team’s experience in Indigenous methods, informed by evolving understanding of two-eyed seeing in Indigenous health research [28] and past efforts to develop two-eyed seeing methodology [29]. Rowan et al. outline their adaptation of the standard six-stage scoping review methodology [30, 31] to include a “base” stage to “assemble an interdisciplinary, inter-professional and intercultural scoping study team” that privileges Indigenous ways of knowing [29]. However, rather than a scoping review approach, we will focus on complementary elements of meta-narrative review methodology within the TESR. Greenhalgh and Wong note that the “meta-narrative approach is intended for those reviews where the underlying research goal is to identify and explore the diversity of research approaches to a topic” [32]. They describe meta-narrative review as a new method of systematic review, designed for topics that have been conceptualized and studied by different groups of researchers. Reviewers consciously step out of their own worldview, learn new vocabulary and methods and try to view a topic through multiple sets of eyes in order to produce an overarching narrative [33]. RAMESES (Realist and Meta-narrative Evidence Syntheses: Evolving Standards) project guidance for meta-narrative reviews outlines nine stages (research problem; understanding and applying the purpose and underpinning principles of meta-narrative reviews; focusing the review; scoping the literature; developing a search strategy; selection and appraisal of documents; data extraction; synthesis phase; and reporting) and a four-point quality scale [33].

Directed by Elders and our steering group, we will braid Gwich’in and Tłı̨chǫ guiding principles with meta-narrative review processes as per the RAMESES project. Drawing on multi-knowing and multidisciplinary expertise from within our research team, our institutions and externally, we will document and unpack at least eight ways of knowing relevant to health system learning, including the following:

1. Indigenous knowledges and ways of knowing, under guidance from our Gwich’in and Tłı̨chǫ partners;
2. traditional healing/medicine;
3. western medicine/clinical epidemiology (e.g. evidence-based medicine, problem-based learning);
4. population and public health;
5. health system strengthening;
6. management and organizational behaviour (e.g. organizational learning, social learning, complex adaptive systems);
7. political/social science;
8. Eastern philosophies.

This work will contribute methodological insights on knowledge synthesis methods that emphasize and privilege Indigenous ways of knowing. Results will be reported in accordance with a modified version of established guidance for meta-narrative reviews [34].

### Phase 2: Multiple case study

Well matched to “how” and “why” questions, multiple case study enables in-depth exploration of specific problems in specific situations [35]. Sometimes misunderstood as a methodological limitation, this respect for the situational nature of knowledge is a strength that permits case study research to examine complex phenomena in context [35, 36]. This also aligns with Indigenous worldviews that see knowledge as place-based [17]. Thus, multiple case study is well suited to studying the interconnectedness between all peoples, processes and things [37, 38]—an important point of compatibility with Indigenous research [17, 39]—and facilitates involvement of Indigenous partners whose contributions will strengthen construct validity and, in this study, help evolve a conceptual framework for understanding health system learning in an Indigenous context. With respect for these contributions, research participants are referred to as *knowledge holders*.

As our conceptual framework develops with contributions from Elders and other knowledge holders over the course of research, specific study propositions may be formulated. For now, there are two key presumptions that will be explored through the multiple case study. First, we presume that relationships between the organization and communities it serves are key to organizational or system learning—possibly as the prompt and even source of learning [13]. Second, we presume that a learning organization that is strong like two people will give rise to a greater range of ways to act, based on a greater range of evidence, values and beliefs.

#### Case selection

GTC, TG and the Government of NT (represented by DHSS, NTHSSA and TCSA) are the three cases in the multiple case study. They were invited to partner on account of their potential to provide rich information, roles in the Indigenous patient experience at various stages of the healthcare continuum and dissimilarities with regard to their respective ways of knowing and relationships with Indigenous communities.

Working with the steering group as well as organizational and community leaders, we are in the process of confirming one Gwich’in and one Tłı̨chǫ community as additional embedded units of analysis. These embedded units within each case will add an additional layer of depth of understanding of the similarities and differences that emerge within organization–community learning relationships, thus anchoring this understanding more firmly within the meso level of the NT health system.

**Fig. 1:**
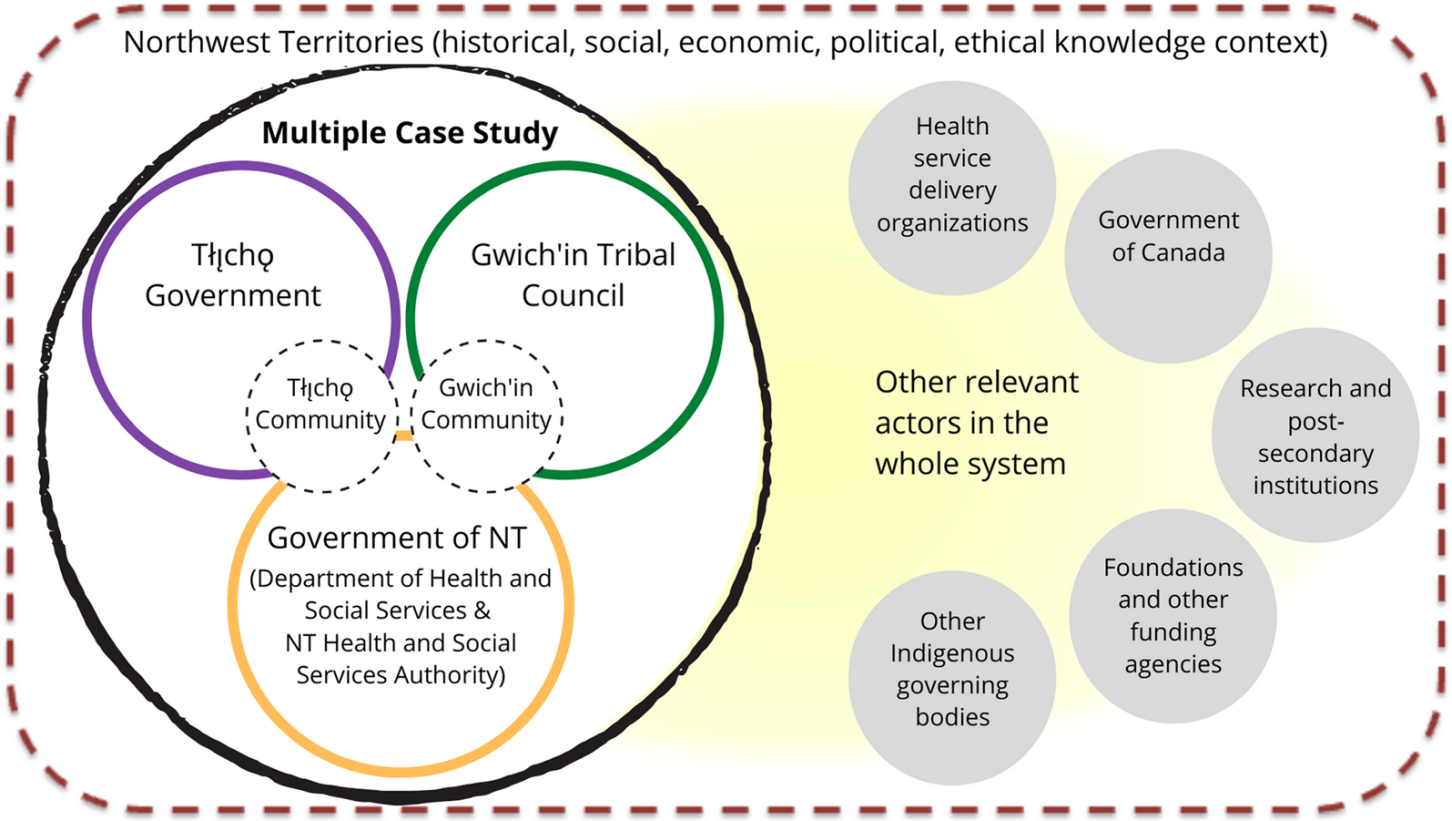
Three organizational cases bounded in the context of Northwest Territories

A case-oriented approach combining in-depth exploration of each case with cross-case comparison will cultivate understanding of the structures and processes that constitute each case as a whole, affording a fuller picture of health system learning than an examination of variables across the cases [40]. The cases and their embedded units of analysis, bounded in the context of other system actors and the broader setting of NT, are visually depicted in Fig. 1.

#### Data collection

The primary methods for data collection will include semi-structured interviews, talking circles, and document analysis. All data will be converged within a case study database managed using NVivo software [41], facilitating the coding of all data (notes, transcripts, documents and preliminary analyses) according to propositions, rival explanations, and emerging themes while establishing an audit trail so that external observers can assess the dependability of research [35, 42].

Semi-structured interviews will enable in-depth exploration with three general knowledge holder categories: organization members, community members and other stakeholders who can speak to the factors and context of organizational learning by the cases. Knowledge holders will be identified through a combined approach of maximum variation sampling, maximizing the diversity of characteristics among organizational and community informants, and snowball sampling wherein participating knowledge holders recommend others we can invite to participate in an interview. Interviews will be held in English for up to 2 hours, either in a private place of the knowledge holder’s choosing or by phone or video conference. Interviews will be recorded and transcribed verbatim. To improve the validity of the research, knowledge holders will be asked to review their transcripts and approve their contributions. Unless acting in an official capacity (i.e. representing an organization where they are a paid employee), knowledge holders will be compensated with a $100 gift card from the local general store. Constant comparison will allow for refinement and re-evaluation of the interview guide and emerging themes throughout data collection, which will continue until the final interview yields no novel findings [43]. We anticipate 15 to 25 interviews per case.

Two talking circles, one in each partner community, will be held when COVID-19 restrictions allow for in-person gatherings. Conducted after preliminary interview data are available, the talking circles will serve as a first round of community-level member-checking. They will generate data (handwritten notes) within a context of relationship and social interconnectedness in communities [39, 44]. We anticipate each circle to run about 4 hours. We will incorporate prayer and ceremony, giving space for wholistic storytelling not fragmented by a structured interview process. To ensure time for storytelling, the circles will be limited to a maximum of 10 knowledge holders representing Elders, youth (aged 18 to 25), officials from the local governing authority, and other groups identified in advance with the steering group. Attempts will be made to include a balanced number of male and female knowledge holders. Each knowledge holder will be given a $100 gift card for participating in the talking circle. Interpretation will be available.

Document analysis can be illuminating, providing insight into systems of social meaning and practice that cannot otherwise be questioned or observed [45]. First, a targeted search strategy will be used to collect documents no older than 10 years that relate to the three organizational cases and their embedded units. Other selection criteria include relevance and contribution to answering the research questions, demonstrated evidence of learning (or not learning) from or with communities, and contribution to conceptualizing a learning organization or learning health system. Second, documents will be obtained via requests to knowledge holders and organizations for additional material. The documents sought will include programme reports and evaluations, meeting agendas, planning documents, policy, written media and other English-language publications. An annotated bibliography of all reviewed documents will be developed and entered into the database.

#### Analytic approach

Data analysis will occur recursively throughout data collection and intensify once all data have been collected. The data will be considered interdependently—categorizing, tabulating or otherwise juxtaposing evidence from different sources to address the study propositions [42]—thus strengthening construct validity [35]. Multilayered reflexivity will add an additional layer of critical evaluation of potential future impact as well as interpersonal and collective dynamics throughout the research process [46, 47].

As part of a two-eyed seeing approach and to reduce bias, preliminary analyses will be conducted through collaboration and consensus development with the steering group. This will occur initially through collaborative reading and analysis of a subset of notes, transcripts and documents, and the development of a coding guide. The research team will then conduct successive iterations of coding as per the constant comparative method [43, 48]. We will take a context analytic approach, recognizing the socially constructed nature of data—particularly for documents intentionally produced for wide distribution [45]. It will therefore be important to examine not just the content of notes, transcripts and documents, but also their sources, and to consider how they reflect organizational or community ways of knowing and learning. The purpose will be not only to provide a rich description of each organization and its context, but also triangulate against other methods and findings.

Positioned by some scholars as a bridge between qualitative evidence and deductive research, case studies can be guided by and generate theoretical propositions [49]. This study will use mixed deductive and inductive coding, drawing on themes from the conceptual framework, themes provided by the steering group, and others inductively generated from the data, privileging Gwich’in and Tłı̨chǫ voice [48]. To enhance credibility, the analysis will include pattern matching (comparing results to study propositions) as well as searching for alternative ways of seeing and understanding the context and each case, so as to rule out rival explanations [35]. Other measures to ensure internal and construct validity (e.g. data and methods triangulation, establishing a chain of evidence, member-checking by knowledge holders, logic models), combined with analysis according to literal and theoretical replication logic, will enhance analytic generalizability and facilitate theory-building on health system learning from different knowledge systems [35, 50].

Each case will be analysed and completed one at a time before cross-case analysis [51]. Organizational partners will be invited to review their draft case report to confirm authenticity and relevance. Cross-case analysis will generate inferences about the dynamic of health system learning from knowledges and evidence held within Indigenous communities. Preliminary findings and conclusions will be shared with knowledge holders for feedback and validation before being finalized. The steering group will also review preliminary results and provide guidance in any instances where there are direct conflicts between different sources of data. The reporting of final results will be guided by our research partners as well as established Standards for Reporting Qualitative Research [52].

### Phase 3: Integrating TESR and multiple case study findings

Phase 3 involves the integration of the findings from the TESR and multiple case study. The TESR will provide a mix of theoretical and empirically derived understandings of health system learning from knowledges and evidence held within Indigenous communities. The multiple case study will provide an empirical lens on the same. To integrate these findings, the main deliverable of phase 3 will be the production of a conceptual model to understand learning organizations and learning health systems. This model will be developed iteratively as we progress through phase 1 and phase 2 and will be a key focus for ongoing discussions with the steering group and other partners.

### Ethics and regulatory approvals

The research team will strictly adhere to national guidance on ethical research involving First Nations, Inuit and Métis peoples living in Canada [53] as well as the principles of ownership, control, access and possession (OCAP^®^) [54]. Before seeking their written informed consent, the research team will provide verbal and written information about the research to all Indigenous and non-Indigenous knowledge holders, who will have the opportunity to ask questions. Anonymity and confidentiality will be protected in final reporting, except in the event Gwich’in or Tłı̨chǫ protocol requires certain knowledge to be attributed to an Elder or other esteemed Knowledge Keeper. In this case, separate additional consent will be obtained. Knowledge holders will retain ownership of their contributions and be offered their respective transcripts or recordings and will have the opportunity to provide input into how their knowledge is disseminated. Pending COVID-19 restrictions, the research team will ensure that findings are presented at in-person community gatherings. Copies of research results, briefs, publications and presentations will be shared with partner communities and organizations.

This study protocol has undergone multiple compulsory reviews. Initial ethical approval was granted by the University of Toronto Research Ethics Board. Two additional rounds of review then had to be completed by the university’s Face-to-Face COVID-19 Review Committee. Coinciding with different waves of COVID-19, the process to obtain all university approvals took longer than 1 year. The Aurora Research Institute thereafter led a review of the study protocol in collaboration with NT community reviewers and granted a license in February 2022 to allow our research team to conduct research in NT. Lastly, this protocol underwent reviews within GTC, TG, DHSS, NTHSSA and TCSA in advance of each organizational partner developing and signing its respective research agreement to govern the project. Each of the steps outlined in this paragraph were necessary before initiating the research.

## Discussion

This study will be valuable across the healthcare sector for its contributions to research, policy and systems building. By documenting and disseminating our efforts to develop two-eyed seeing research methods, bringing different groups together to examine evidence from multiple ways of knowing, we will contribute to the establishment of strengths-based research methodology that can be applied to the most acute and complex challenges to health equity.

This study will inform efforts to embed learning within healthcare systems and shed light on barriers and facilitators in the respectful incorporation of community knowledges into evidence structures and processes. Privileging Gwich’in and Tłı̨chǫ perspectives and knowledges throughout the research will help to identify what, from a patient and community perspective, health systems must learn in order to do better by all. Our results will inform a comprehensive two-eyed seeing framework for conceptualizing evidence from which health systems can learn, pointing them toward valuable evidence for culturally safe, patient-centred decision-making.

Given the lack of consensus on a definition for a learning health system, and despite its inconsistent usage as a term, our focus on organizational learning will not only enhance and deepen definitions of learning organizations and learning health systems—making them more practicable—but also have clear implications for our understanding of broader systems of organizations and other entities. Although our primary focus is on organizations and communities at the meso level, the implications of this research could influence all levels of healthcare. Notably, we anticipate this study will inform individuals as well as whole systems in tailoring their approaches to the contexts they are in. Just as they may encourage individuals and units in organizations to strengthen their relationship-centred practice, our findings will point organizations toward equitable policy, processes and structures for meaningful interactions and learning with the communities they serve.

Examining the systems that create health disparities has become an imperative for Canadian healthcare. This study will contribute to meeting this imperative by supporting healthcare organizations and systems to listen and learn, thus broadening our frame of what is valued as knowledge and illuminating a path to reconciliation in Canadian healthcare.

## Data Availability

Not applicable.

## Abbreviations

COVID-19: Coronavirus disease 2019
DHSS: Department of Health and Social Services
FRN: Funding reference number
GTC: Gwich’in Tribal Council
NT: Northwest Territories
NTHSSA: Northwest Territories Health and Social Services Authority
OCAP^®^: Ownership, control, access and possession
RAMESES: Realist and Meta-narrative Evidence Syntheses: Evolving Standards
TCSA: Tłı̨chǫ Community Services Agency
TESR: Two-eyed seeing review
TG: Tłı̨chǫ Government
